# Green Radish Polysaccharide Prevents Alcoholic Liver Injury by Interfering with Intestinal Bacteria and Short-Chain Fatty Acids in Mice

**DOI:** 10.3390/foods13233733

**Published:** 2024-11-22

**Authors:** Xiong Geng, Miaomiao Zhuang, Weina Tian, Huayan Shang, Ziyi Gong, Yanfang Lv, Jianrong Li

**Affiliations:** College of Food Science and Engineering, Bohai University, Jinzhou 121013, China; 18748135846@163.com (X.G.); zmm13865981106@163.com (M.Z.); kjcy10@163.com (W.T.); shy2023015150@bhu.edu.con (H.S.); xiaoxiong314@163.com (Z.G.); lvyanfang2003@126.com (Y.L.)

**Keywords:** alcoholic liver disease, green radish, gut microbiota, polysaccharide, short-chain fatty acid metabolism

## Abstract

This study aimed to ascertain the potential benefits of green radish polysaccharide (GRP) in treating alcoholic liver disease (ALD) in mice and explore its mechanism of action. Using biochemical analysis, high-throughput sequencing of gut microbiota, and gas chromatography–mass spectrometry to measure short-chain fatty acids (SCFAs) in feces, we found that GRP intervention significantly improved lipid metabolism and hepatic function in mice subjected to excessive alcohol intake. The GRP intervention reduced malondialdehyde levels by 66% and increased total superoxide dismutase levels by 22%, thereby mitigating alcohol-induced oxidative stress. Furthermore, GRP intervention in mice with alcohol consumption resulted in a reduction in tumor necrosis factor, interleukin 6, and lipopolysaccharide levels by 12%, 9%, and 25%, respectively, effectively attenuating alcoholic liver inflammation. 16S rRNA amplicon sequencing demonstrated that excessive alcohol consumption markedly altered the gut microbiota composition in mice. The GRP treatment resulted in a significant reduction in the number of beneficial bacteria (*Lactobacillus* and *Lachnospiraceae_NK4A136_group*) and an increase in the proportion of harmful bacteria (*Muribaculaceae* and *Verrucomicrobiota*). The metabolomic analyses of the SCFAs demonstrated an increase in the contents of SCFAs, acetic acid, propionic acid, and butyric acid, following GRP supplementation. Furthermore, the metabolic levels of cholinergic synapses and glycolysis/gluconeogenesis were found to be modulated. In conclusion, these findings suggest that GRP may attenuate alcohol-induced oxidative damage in the liver by modulating the gut microbiota and hepatic metabolic pathways. This may position GRP as a potential functional component for ALD prevention.

## 1. Introduction

Alcoholic liver disease (ALD) occurs when acute or chronic alcohol abuse damages the liver [1]. It initially presents as marked hepatocyte steatosis, progressing to fatty liver, fibrosis, and cirrhosis. Heavy alcoholism over the short term can also cause acute-on-severe alcoholic hepatitis, acute-on-chronic liver failure, and even death [2,3]. ALD is the second most common liver condition (affecting approximately 0.6 billion people) in China, surpassed only by viral hepatitis (affecting approximately 2 billion people) [4]. About 80% of blood alcohol is metabolized in the liver, producing reactive oxygen species that damage liver cells [5,6]. Oxidative stress plays a key role in ALD progression [7]. Normally, the oxidative and antioxidant systems of vertebrates are in dynamic equilibrium, “oxidative stress” occurs when the delicate biochemical balance between the oxidative and antioxidant systems is disrupted [8]. The consumption of large amounts of alcohol over a long time leads to the depletion of the enzyme alcohol dehydrogenase, which is essential for alcohol metabolism. The microsomal oxidase system then performs oxidation of alcohol into acetaldehyde. Oxidative stress is responsible for the mitochondrial dysfunction, steatosis, inflammation, and fibrosis induced by ethanol [9]. More than 150 million people suffer from ALD globally, with incidence rates continuing to rise [10]. Commonly used drugs for treating ALD include hydroxy phosphatidylcholine, silymarin, and glucocorticoids. However, most drugs have some adverse side effects [11,12]. Polysaccharides from natural products are an excellent source of antioxidants. For example, Chen Feng et al. reported the antioxidant properties of *Momordica charantia* polysaccharide [13], Hou Ranran et al. found that *Echinacea purpurea* polysaccharides exerted antioxidant effects by inhibiting apoptosis and promoting the Nrf2 cell signaling pathway [14]. Therefore, exploring natural products with potent antioxidant activity from food sources is important for preventing ALD.

Emerging evidence indicates potential roles of the gut microbiome and SCFAs in ALD pathogenesis [15,16]. The gut microbiota plays a crucial role in the host’s energy metabolism. Alcohol consumption may indirectly contribute to liver damage by disrupting the gut microbiota and increasing intestinal permeability. This can lead to the translocation of gut bacteria and increased plasma bacterial endotoxin (enterogenous lipopolysaccharide, LPS) levels [17,18,19]. The influx of lipopolysaccharide is controlled by Toll-like receptors on the cell surface, which initiate cytokine production of interleukin (IL)-6 and tumor necrosis factor (TNF)-α and induce low-grade inflammation, contributing to the initiation and development of obesity and its associated complications [20]. Additionally, LPS has been demonstrated to affect gut microbiota composition and function [21]. SCFAs are derived from the fermentation of dietary fiber by the gut commensal microbiota, providing an energy source for intestinal epithelial cells and regulating hepatic glucose and energy metabolism [22,23,24], and they are thought to be essential signals in the liver–intestinal axis. SCFAs, which may contribute to the prevention of liver disease and the regulation of metabolism, may also regulate lipid metabolism and immune-response-associated gene expression and contribute to many facets of liver disease, including steatosis, hepatitis, inflammation, and hepatocellular carcinoma. Supplementation with SCFAs improves intestinal permeability and inhibits HDAC1 expression to attenuate hepatic steatosis and injury [25,26,27,28]. In human studies, reductions in SCFA concentrations, including acetic and propionic and butyric acids, were observed in patients with severe alcoholic hepatitis [22]. Prebiotics such as xylooligosaccharide (XOS) increase the abundance of beneficial bacteria such as *Bifidobacterium* and *Lactobacillus* [29], Inonotus hispidus exopolysaccharides prevent acute ALD [30], and *Dendrobium huoshanense* polysaccharides restore the perturbed metabolism pathways following ethanol exposure to prevent the progression of ALD [31]. Jiang Wenhao et al. examined *Echinacea purpurea* polysaccharide, the biochemical indicators of liver function, histopathological sections, and mRNA and protein expression of relevant antioxidant enzymes in ALD [32].

Green radish (*Raphanus sativus* L.) is a variety of radish with a pleasant taste, which is favored by consumers and is known as “underground ginseng”. The nutritional value of green radish easily meets quality requirements because of its short growth cycle. Polysaccharide is an important component of radish with many physiological functions. In previous studies (“Optimisation of green carrot polysaccharide extraction process and analysis of antioxidant activity based on enzymatic hydrolysis method”, accepted by *Modern Food Science and Technology*, in press), the major polysaccharides isolated from green radish by our team using enzymatic methods were mannose, ribose, rhamnose, gluconate, galacturonic acid, glucose, galactose, xylose, arabinose, and fucose (Figure S1). The strong absorption peak of green radish polysaccharide (GRP) at 3440.81 cm^−1^ is attributed to the O–H stretching vibration, whereas the peak observed at 2927.04 cm^−1^ is ascribed to the C–H stretching vibration. The peak at 1633.3 cm^−1^ is associated with the C=O vibration of the carbonyl group, whereas the peak at 1016.56 cm^−1^ indicates stretching vibrations of C–O. These peaks are characteristic of sugars. Additionally, the peak at 599.08 cm^−1^ is attributed to the O–H surface bending vibration (Figure S2). Our study reported the role of GRP, a plant polysaccharide, in gut health and obesity (submission in progress). However, the protective impact of GRP on liver tissue and the metabolic profile of GRP in liver tissue are still unknown.

This study was performed to investigate the beneficial effects of GRP on ALD and its underlying mechanisms using histopathology, biochemical analysis, 16S rRNA analysis, and targeted metabolomics. The results show that GRP attenuated ALD by regulating gut microbiota and thereby inhibiting hepatic lipid oxidation signaling pathways. This study provides new insights into the prevention and treatment of liver disease by providing a mechanistic explanation of the function of the gut–liver axis in attenuating ALD.

## 2. Materials and Methods

### 2.1. Biological Methods

Green radish and 56% (*v*/*v*) Niulanshan Erguotou white spirit were purchased from Jinzhou General Supermarket (Jinzhou, China). Kunming (KM) mice were supplied by Jinzhou Aohong Pharmaceuticals Co., Ltd. (Jinzhou, China).

### 2.2. Major Instruments and Equipment

An FW177 pulverizer (Tianjin Tester Instrument Co., Ltd., Tianjin, China); UV-2550 ultraviolet-visible spectrophotometer (Shimadzu (Guangzhou) Testing Technology Company Limited, Guangzhou, China); RE-52AA rotary evaporator (Shanghai Yarong Biochemical Instrument Factory, Shanghai, China); Scientz-10N vacuum freeze dryer (Ningbo Xinzhi Bio-Technology Co., Ltd., Ningbo, China); Biofuge Stratos frozen high-speed centrifuge (Thermo Fisher Scientific (China) Limited, Shanghai, China); SHB-IIIG Circulating Water Multi-Vacuum Pump (Zhengzhou Century Shuangke Experimental Instrument Co., Ltd., Zhengzhou, China); SG2-ELK Portable pH Meter (Mettler Toledo Technology (China) Co., Ltd., Shenzhen, China); LC-20AD High-Performance Liquid Chromatography (Shimadzu Enterprise Management (China) Co., Ltd., Shanghai, China); iS50 Infrared Spectrometer (Nicholi Instruments, Inc., Shanghai, China); and GPC-20A Gel Permeation Chromatograph (Shimadzu Corporation).

### 2.3. Preparation of GRP

The main extraction methods for polysaccharides are water leaching [33], ultrasonic-assisted extraction [34], microwave-assisted extraction [35], enzyme-microwave-assisted extraction [36], and low-pressure extraction [37]. The composition of polysaccharides may be influenced by the extraction method employed. Ultrasonic-assisted extraction was used in this study because of its ability to reduce the extraction time, increase the polysaccharide yield, and maintain the biological activity, as well as enhance the recovery of some fractions that are less readily accessible.

The leaves and roots of green radish were removed, sliced, dried in a low-temperature oven at 37 °C, crushed, and sifted through a 40-mesh sieve. Then, 50 g of radish powder was dissolved in ethanol at a ratio of 1:5, placed in a water bath at 50 °C for 8 h, filtered using gauze, and the supernatant was discarded [38]. Referring to Wang Fei et al.’s response surface methodology to optimize the extraction of radish polysaccharides and the exploration of their in vitro antioxidant activity [39], the residue after filtration was extracted with distilled water at a ratio of 1:25. This was followed by ultrasound-assisted hot water extraction twice (at 50 °C and 230 W for 29 min), filtration, and centrifugation. The supernatant after centrifugation (two times) was combined, subjected to rotary evaporation, and concentrated to 20% of the original volume. Further, the supernatant was centrifuged (4500 rpm for 15 min) and precipitated with ethanol (3:7, *m*/*v*). The precipitate was centrifuged at 3000 rpm for 35 min, redissolved in water, and dried at low temperature, yielding 1.4 g of polysaccharide [38].

The Sevage method for protein removal is as follows: Crude polysaccharide was prepared at a mass concentration of 1 mg/mL, Sevage reagent was added at the ratio of 1:5:25 for *v* (n-butanol):*v* (trichloromethane):*v* (crude polysaccharide), shaken for 20 min, and then centrifuged for 15 min at 4500 rpm to remove the supernatant. This process was repeated three times until no protein layer remained. Then, the supernatant was centrifuged and concentrated, followed by alcohol precipitation and freeze-drying to obtain GRP [40]. The absorption peaks at 280 and 260 nm were observed using an ultraviolet (UV) spectrophotometer, the composition and molecular weight of monosaccharides were determined by high-performance liquid chromatography (HPLC) [41], and GRPs were analyzed using a Fourier-transform infrared (FT-IR) spectrometer in the wavelength range of 4000–500 cm^−1^ [42].

### 2.4. Animal Experiments

Animal experiments were approved by the Animal Experimentation Centre Ethics Committee of Jinzhou Medical University (Jinzhou, China, SCXK (Liao) 2019-0003). Specific-pathogen-free (SPF) KM mice (male, aged 4–5 weeks, weighing 18–22 g) were housed at the Jinzhou Medical University Animal Experimental Centre. All mice were maintained at a relative temperature of 21–25 °C, a relative humidity of 60–65%, and a light–dark cycle of 12–24 h.

All mice were divided into the following three groups (12 mice/group): control group (saline), model group (56% (*v*/*v*) Niulanshan Erguotou white spirit administration, 12 mL/kg/d), and GRP (400 mg/kg/d) [43]. First, the control and model groups received the same amount of saline gavage every day, and the GRP group received the same amount of polysaccharide gavage every day. Subsequently, after 30 min elapsed, the control mice were gavaged with an equal volume of saline, and the mice in the model and GRP groups were gavaged with 12 mL/kg of 56% (*v*/*v*) Niulanshan Erguotou white spirit to establish an acute liver injury model. Alcohol and GRP solutions were administered via gavage continuously for 3 weeks following the aforementioned methodology. After 12 h of fasting, blood was taken from the eyeballs and centrifuged to obtain the supernatant, and the mice were dissected after cervical dislocation. The liver was quickly removed and stored in a refrigerator at −80 °C for subsequent index determination.

### 2.5. Hematoxylin and Eosin Staining

The liver samples were fixed with 4% formaldehyde, immersed in an ethanol series and xylene, embedded in paraffin, and sectioned at 5 μm. The histopathological changes were visualized using hematoxylin–eosin (H&E) staining and standard light microscopy [44].

### 2.6. Serum and Liver Biochemical Marker Assays

Serum biochemical markers, including total cholesterol (TC), triglyceride (TG), aspartate aminotransferase (AST), and alanine aminotransferase (ALT), were determined using corresponding commercially available kits (Nanjing Jianjian Co., Ltd., Nanjing, China) and following the manufacturer’s protocol. Then, 0.2 g of liver tissue was homogenized in nine times 0.86% saline solution and then centrifuged at 3000 rpm for 15 min at 4 °C. The levels of catalase (CAT), malondialdehyde (MDA), total superoxide dismutase (T-SOD), and glutathione peroxidase (GSH-Px) were determined using corresponding kits. Serum lipopolysaccharide (LPS), interleukin-6 (IL-6), and tumor necrosis factor-α (TNF-α) levels were determined at 405 nm using a mouse enzyme-linked immunosorbent assay kit and following the manufacturer’s protocol.

### 2.7. Amplicon Sequencing and Bioinformatics Analysis

After 3 weeks, fresh fecal samples (control group, model group, and GRP group; *n* = 6) from separately caged mice were collected in 2 mL sterilized tubes and stored at −80 °C. DNA was extracted from the mice feces, and the quality of the extracted DNA was checked using a DNA kit and following the manufacturer’s protocol. Polymerase chain reaction (PCR) was used for amplification based on the V3–V4 area of the 16S rDNA. The PCR amplicons were purified using AMPure XP beads (Beckman Coulter, Shanghai, China) and quantified using the PicoGreen dsDNA detection kit (Invitrogen, Carlsbad, CA, USA). The Illumina NovaSeq6000 platform was used to perform gene sequencing on mouse fecal samples. Raw sequences were screened using an in-house program, and the pairs were assembled using Vsearch V2.4.4 (-fastq_mergepairs-fastq_minovlen 5). Operational taxonomic units (OTUs) were selected using vsearchv2.15.0 prior to the 16S rDNA data analysis. The OTU and genome sequence data were processed using the Quantitative Indications in Microbial Ecology (QIIME2, v2020.6) framework. This involved chimera de-replication (--derep_fulllength), clustering (--cluster_unoise), and detection (-uchime3_denovo) [45]. The data were visualized using heat maps and networks in R software (version 3.3.2) and Cytoscape (version 3.9.0), as appropriate. The metabolic function of the gut microbiota was predicted by PICRUSt, which can predict the 16S rRNA gene sequence in the KEGG functional spectrum database. According to the forecast results of PICRUSt, annotation information from each functional spectrum database could be obtained for each sample and the functional category of the abundance matrix predicted. A violin plot was then drawn to display the result.

### 2.8. Analysis of Short-Chain Fatty Acids in Feces Using Metabolomics and Gas Chromatography–Mass Spectrometry

After 3 weeks, fresh fecal samples (control group, model group, and GRP group; *n* = 6) from separately caged mice were collected in 2 mL sterilized tubes and stored at −80 °C. The concentrations of various acids, including acetic acid, propionic acid, butyric acid, hexanoic acid, isobutyric acid, valeric acid, and isovaleric acid, in feces were measured using gas chromatography–mass spectrometry (GC-MS) [46]. To prepare the samples, 20 mg of feces were homogenized and diluted in ultrapure water using a microtube homogenizer. The resulting mixture was sonicated for 15 min and then centrifuged at 10,000 rpm and 4 °C for 10 min to obtain the supernatant. Then, 200 μL of this mixture was added to 20 μL of 4-methylpentanoic acid as an internal standard and derivatized with 100 μL of phosphate-buffered saline and 280 μL of ether. After centrifugation at 10,000 rpm for 10 min, the upper organic layer was transferred to a sample vial and analyzed using a TRACE 1300 gas chromatography–mass spectrometer (Thermo Fisher Scientific, Waltham, MA, USA). The GC system’s conditions included the installation of an HP-5 capture tower (30 m × 0.25 mm × 0.25 μm) using argon as the carrier gas [47,48]. The splitting ratio of the injection was 30:1, the injection volume was 1 μL, and the operating temperature was 250 °C. The processing conditions were as follows: oven temperature of 70 °C, held for 3 min, then increased to 130 °C at a rate of 10 °C/min, and thereafter to 280 °C at a velocity of 30 °C/min, and, finally, held for 2 min. The mass spectrometric system was operated under the following conditions: ion source temperature of 230 °C, transmission line temperature of 290 °C, EI ionization mode with electron energy of 70 eV, and a solvent delay of 7 min. Scanning was performed in the selected ion monitor mode. Short-chain fatty acids (SCFAs) were quantified using the corresponding calibration curves. After obtaining the original data, Progenesis QI v2.3 software was used to perform standardized preprocessing and qualitative and relative quantitative analyses.

### 2.9. Statistical Analysis

The data are expressed as the mean ± standard deviation and statistically analyzed using SPSS 22.0 and Origin 19.0 program. GraphPad Prism (ver. 8.0) software was used to perform one-way analysis of variance. Differential metabolite clustering heat map normalization (Z-score) of differential metabolites among groups was used for the cluster analysis. A heat map function in the R language was used to visualize the differences in the accumulations of differential metabolites among groups; * *p* < 0.05 and ** *p* < 0.01.

## 3. Results and Discussion

### 3.1. Composition of GRP

The GRP was characterized using FT-IR in this study’s spectroscopic and molecular weight analyses, with the results aligning with our previous research, confirming its identity. The GRP was subjected to UV spectroscopy. No absorption peaks were observed at 260 and 280 nm (Figure S3). The molecular weight of the GRP was divided into four fractions, with weights of 4.822 kDa, 25.538 kDa, 634.808 kDa, and 1.342 KDa (Figure S4).

### 3.2. Effect of GRP on Weight and Organ Index

**Figure 1:**
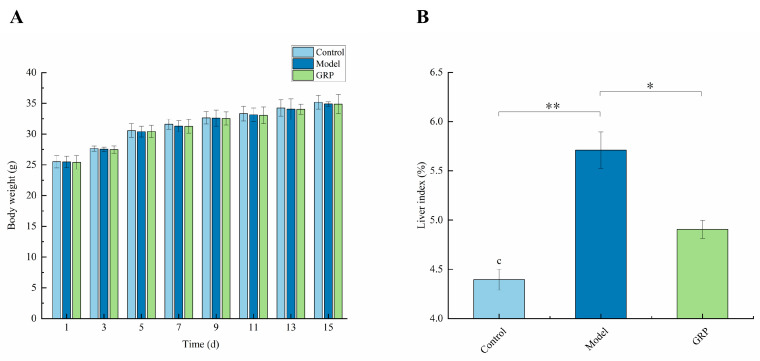
GRP did not affect body weight in alcohol-induced mice but decreased alcohol-induced organ index: (**A**) representative weights; (**B**) organ index. The results are expressed as the mean ± SD (*n* = 6–8). * *p* < 0.05 and ** *p* < 0.01.

The body weights were not significantly different among the control, model, and GRP groups (Figure 1A), suggesting that GRP had no impact on the growth of mice. The indicator of liver pathology [49] had obviously higher values in the model group than in the control group (Figure 1B). The study showed that the successful establishment of the model was influenced by the fact that alcohol caused edema and damage to liver tissue. The liver index significantly decreased in the GRP group (*p* < 0.05) compared with the model group, suggesting that GRP effectively reduced liver edema and acute ALD. Specifically, the GRP intervention significantly reduced the liver index (up to a 16% reduction).

### 3.3. Liver Tissue Morphology and Serum Biochemical Markers of Liver Injury

**Figure 2:**
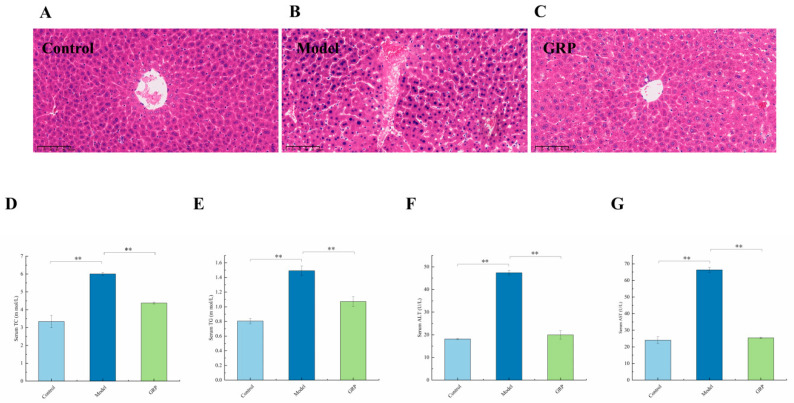
Effects of GRP on serum biochemical indices and histopathology. (**A**–**C**) Representative images of hematoxylin and eosin (H&E) staining of liver tissues. The images are shown at a 20× zoom level; the ratio was 100 μm. (**D**–**G**) The levels of TC, TG, ALT, and AST in serum were measured, and the results are expressed as the mean ± SD (n = 6–8). ** *p* < 0.01.

This study reveals altered hepatic morphology among the mice from different groups. The histopathological results show that liver cells in the control group (Figure 2A) had a normal morphology with homogeneous cytoplasm and clear nuclei. However, the liver cells around blood vessels were disorderly in their arrangement, the hepatic sinusoids were atrophic, and the cytoplasm of liver surface cells was loose in the model group (Figure 2B). The histopathological results show that the size of liver sinusoids was normal in the GRP group compared with the model group. Liver cells were arranged regularly (Figure 2C). This suggests that GPR ameliorated ALD to some extent.

The presence of ALD is associated with elevated levels of serum metabolites and lipid metabolism. TC and TG serve as crucial indicators of liver injury because their increased levels lead to the excessive accumulation of liver fat or impaired metabolism [50]. The mice in the model group exhibited increases of 44% and 46% in the serum TC and TG levels, respectively, compared with those in the control group. Supplementation with GRP effectively mitigated the abnormal elevation caused by excessive alcohol consumption, resulting in reductions of 27% and 28% in the TC and TG levels, respectively, among mice in the GRP group (Figure 2D,E). Additionally, GRP decreased the levels of AST and ALT in serum, thereby improving liver cell injury induced by membrane damage and the subsequent release of aminotransferases into circulation [51,52,53]. Oxidative stress occurs in the liver under the stimulation of alcohol metabolism, damaging liver cells. AST and ALT are highly specific indicators of liver cell damage. Dong et al. [51] found that the levels of AST and ALT were high in healthy liver but low in blood. Once liver cells are damaged, ALT and AST are released in large amounts into the serum to increase serum contents. AST and ALT levels indicate the level of hepatocyte injury in some areas. Reddy et al. [50] discovered higher serum ALT and AST levels in the model group compared with the control group, with increases of 62% and 64%, respectively, indicating that alcohol significantly elevated the levels of these markers among mice (*p* < 0.01). However, the administration of GRP led to decreases of 58% and 62% in the serum ALT and AST levels, respectively, in the GRP group (Figure 2F,G), suggesting alleviating effects of GRP on ALD (*p* < 0.01).

A decrease in the liver index was witnessed in the GPR group. Furthermore, the serum TC, TG, AST, and ALT levels in the model group confirmed liver injury. In contrast, those in the GPR group were reduced, indicating that GPR prevented acute ALD in mice.

### 3.4. Biochemical Markers of Liver Injury

**Figure 3:**
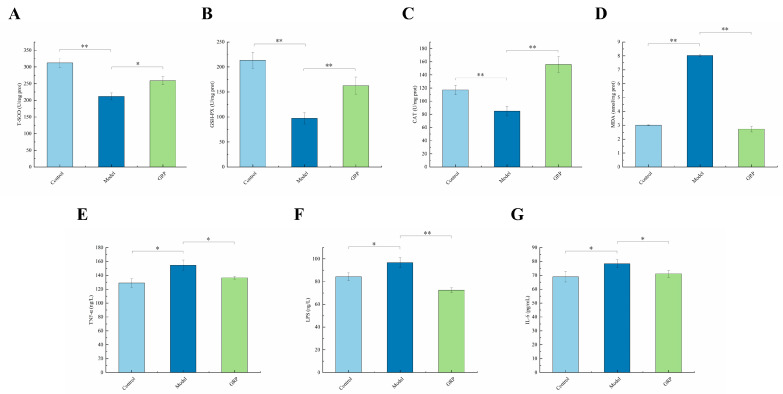
The levels of T-SOD, GSH-Px, MDA, CAT, TNF-α, LPS, and IL-6 in the liver tissues of each group were measured (**A**–**G**), and the results are expressed as the mean ± SD (*n* = 6–8). * *p* < 0.05 and ** *p* < 0.01.

ALD induces oxidative stress and lipid peroxidation and reduces the activities of enzymes involved in protection against injury [51]. The activities of T-SOD and GSH-Px are important in quenching reactive oxygen species. When T-SOD and GSH-Px activities are reduced, the number of free radicals increases, damaging lipids, proteins, and nucleic acids. Excessive alcohol consumption can reduce the activity of antioxidant enzymes in the liver, disrupt the body’s oxidative–antioxidative balance, induce oxidative stress, and trigger lipid peroxidation, mitochondrial dysfunction, endoplasmic reticulum stress, and a chain reaction of liver damage caused by immune inflammation. As we all know, free radicals are easily scavenged by GSH (glutathione), which has active sulfhydryl groups with oxidative dehydrogenation potential. Under the catalysis of T-SOD, GSH-Px, and CAT, superoxide radicals are decomposed into water and oxygen, which are harmless to the body [54]. We found that GRP supplementation significantly enhanced the effects of GSH-PX and T-SOD (Figure 3A,B). The activities of GSH-PX and T-SOD were significantly increased by 67% and 22%, respectively, upon adding GRP (Figure 3A,B). In addition, GRP reversed the ALD-induced increase in the hepatic MDA concentration and the decrease in the CAT concentration, thus improving the redox balance. The MDA concentration decreased by 66% and the CAT concentration increased by 84% after adding GRP (Figure 3C,D). GRP alleviated oxidative damage in the model group. We observed that the model group exacerbated the reduced activity of antioxidases. GRP increased the enzyme activities of SOD, GSH-Px, and CAT and alleviated the lipid and protein oxidative damage in ALD-induced mice. MDA is a product of lipid peroxidation, which can seriously damage the cell membrane, lipoprotein, and other lipid-containing structures, such as by changing the membrane fluidity and permeability, as well as by damaging DNA and proteins, thus affecting normal cell function [55]. In our study, GRP led to reductions in oxidative stress and lipid peroxidation and an improvement in antioxidant enzyme activities. Therefore, we speculate that GRP has the potential to alleviated oxidative damage, which may help to improve intestinal barrier integrity and inflammation.

ALD is related to elevated levels of inflammatory cytokines [56]. Figure 3 shows that the serum TNF-α level of mice increased significantly by 17% in the model group compared with the control group. Additionally, the serum levels of TNF-α were markedly reduced in the GRP group (*p* < 0.05) (Figure 3E). However, intestinal disorders in animals administered alcohol may affect intestinal permeability and inhibit lymphocyte proliferation and differentiation, thereby leading to the entry of bacteria into circulation [57] and subsequent occurrences of ALD and related diseases. In this study, we examined the effects of ALD on serum LPS and IL-6 levels. We found that the serum concentrations of LPS and IL-6 increased by 13% and 12%, respectively, in the model group compared with the control group, and the concentrations of LPS and IL-6 after GRP consumption decreased by 25% and 9%, respectively, compared with the GRP group (Figure 3F,G). When the intestinal integrity was impaired, LPS, bacteria, and other metabolites produced by the pathogenic intestinal flora went through the intestinal epithelia, eventually inducing abnormal expressions of cytokines [58]. Not surprisingly, a much higher level of serum LPS was observed in the model group. Supplementation with GRP significantly reduced the level of LPS compared with the model group. Consistent with the effect of alleviating oxidative stress, GRP significantly suppressed the overexpression of TNF-a, IL-6, and IL-1β in contrast to the model group.

GRP increased the antioxidant enzyme activity and improved lipid peroxidation levels in mice with ALD. It also inhibited elevations in serum TNF-α, IL-6, and LPS levels and reduced cellular inflammation, resulting in therapeutic effects in these mice.

### 3.5. Effect of GRP on Intestinal Microbial Structure

**Figure 4:**
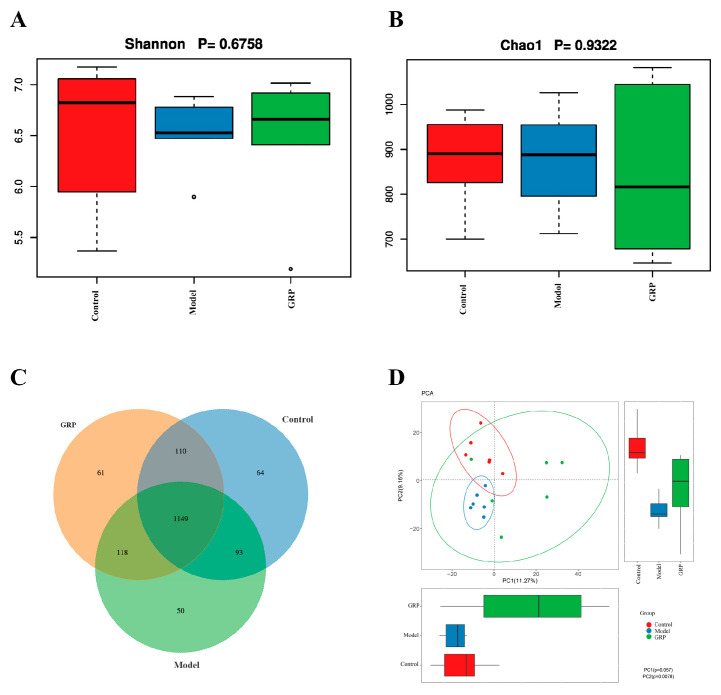
GRP altered gut microbial composition and diversity in alcohol-induced mice: (**A**) Shannon index; (**B**) Chao1 index; (**C**) Venn diagram; (**D**) PCA diagram.

As gut flora play a crucial role in the breakdown of dietary indigestible carbohydrates, fecal samples were collected to analyze the effect of GRP on the intestinal flora. The results of the alpha diversity index showed that the Chao1 and Shannon indices were lower in the model group than in the normal group (Figure 4A,B). The results indicate that the diversity of the cecum gut microbiota was reduced in the model group. Venn plots showing unique and common OTU data (Figure 4C) show that 1149 OTUs were common among all three groups, with 61 unique OTUs in the normal, 64 in the model, and 50 in the GRP groups. Further, 118 OTUs in the GRP group were also present in the control group (Figure 4C). The principal component analysis revealed a considerable difference in similarity between the model and control groups. However, the administration of GRP substantially altered the composition of the community influenced by excessive alcohol consumption (Figure 4D). These conclusions suggest that GRP supplementation might regulate the composition of the gut microbiota and ameliorate the diminished abundance and variability in the community caused by excessive alcohol consumption.

### 3.6. GRP Improved Over-Drinking-Induced Microbiota Dysbiosis at Different Levels

The relevance of the gut microbiota in the pathogenesis of chronic liver conditions has received increasing attention with the growth in our understanding of the enterohepatic axis in the last several years. Alcohol can cause gastrointestinal disturbances, leading to an increase in the abundance of Gram-negative bacteria [59,60], reduction in the number of SCFA-producing cells [61], increase in the abundance of Gram-negative bacteria [62], and upregulation of the hyperpermeability of the intestinal mucosa.

**Figure 5:**
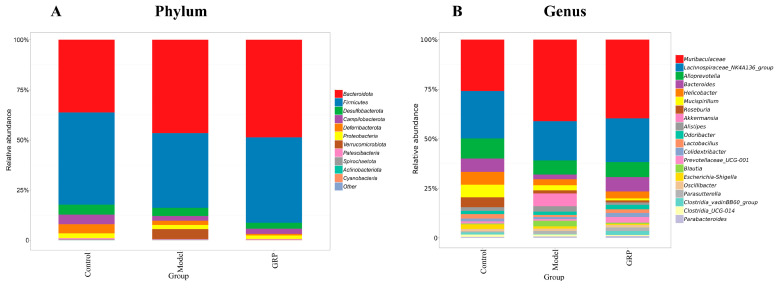
GRP altered the composition of the gut microbiota in alcohol-administered mice: (**A**) average relative abundance of each group at the gate level; (**B**) average relative abundance (*n* = 6) for each genus level.

At the phylum level (Figure 5A), the abundance of *Firmicutes* decreased, whereas the abundance of *Bacteroidota* and *Verrucomicrobiota* increased in the model group compared with the control group. Yan et al. [63] found that the homeostasis of the bacterial populations in the gastrointestinal tract was disturbed in mice administered ethanol for 3 weeks. *Firmicutes* was less abundant, but *Bacteroidetes* and Gram-negative bacteria were more abundant [7]. In 1984, Bode investigated the alterations in the gut microbiota of patients suffering from hepatic alcoholism and found that the abundance of anaerobic and aerobic Gram-negative bacteria significantly increased in patients with hepatic alcoholism [64]. The abundance of *Firmicutes* increased, the abundance of *Verrucomicrobiota* decreased, and the abundance of *Bacteroidota* increased in mice administered alcohol and supplemented with GRP.

At the genus level (Figure 5B), *Muribaculaceae* (26%), *Lachnospiraceae_NK4A136_group* (20%), *Alloprevotella* (10%), *Bacteroides* (7%), and *Helicobacter* (5%) dominated the intestinal flora in the control group. The abundance of *Muribaculaceae* was significantly higher (55%) in the model group compared with the control group. Li Hailong et al. [15] examined Rosa rugosa polysaccharides and found that the occurrence of ALD in mice was consistently associated with the presence of *Muribaculaceae*. The abundance of *Muribaculaceae* decreased upon administering R. rugosa polysaccharide to mice with ALD. The abundance of *Muribaculaceae* decreased after adding GRP. Changes in the abundance of *Muribaculaceae* can contribute to a loss of barrier integrity in the gut, thereby increasing the risk of cancer [29]. Regulating the richness of *Muribaculaceae* can improve conditions such as inflammation and obesity [30]. The levels of *Lactobacillus* and *Lachnospiraceae_NK4A136_group* increased in the control group (49% and 12%, respectively) compared with the model group (*p* < 0.05). As the major probiotic in the intestine, *Lactobacillus* is beneficial for host health. It may effectively improve liver inflammation and intestinal leakage in patients with ALD and may also have a protective effect against ALD [59]. The abundance of Lactobacillus significantly increased (64%) in the GRP group compared with the model group (*p* < 0.05). In summary, the results of the GRP treatment indicated an increase in the abundance of Firmicutes, Lactobacillus, and *Lachnospiraceae_NK4A136_group*, which is beneficial to the stability of the gut microbiota.

### 3.7. Metabolic Analysis of the Hepatoprotective Effect of GRP

**Figure 6:**
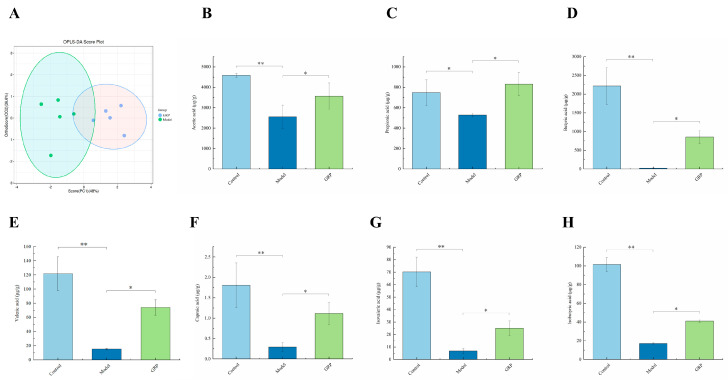
Fecal metabolomics LC-MS analysis. (**A**) The OPLS-DA rating scale shows the differences in metabolites among the groups. The horizontal coordinate indicates the inter-group change, and the vertical coordinate indicates the intra-group change. (**B**–**H**) The acetic acid, propionic acid, butyric acid, valeric acid, caproic acid, isovaleric acid, and isobutyric acid levels are expressed as the mean ± SD (*n* = 6). * *p* < 0.05 and ** *p* < 0.01.

Orthogonal partial least squares–discriminant analysis (OPLS-DA) was used to identify metabolites from the focused metabolomics data. A scatter plot analysis revealed a notable upward trend in the abundance of *Lactobacillus* and *Lachnospiraceae_NK4A136_group* in the control group (49% and 12%, respectively) compared with the model group (Figure 6A).

The changes in the microflora of the fecal samples show that GRP reduced the abundance of *Muribaculaceae* and *Verrucomicrobiota* (Figure 5A,B). The abundance of *Lactobacillus*, *Lachnospiraceae_NK4A136_group*, *Firmicutes*, and *Bacteroidota* increased. A reduction in the abundance of *Lactobacillus* led to decreases in the SCFA concentrations, as demonstrated by Yao Du et al. [64]. We analyzed mice fecal samples using a targeted metabolomics GC-MS approach to examine the impact of SCFAs on the development of ALD. The analysis revealed the presence of seven SCFAs, namely, acetic acid, propionic acid, butyric acid, valeric acid, hexanoic acid, isovaleric acid, and isobutyric acid, in the excrement of all mice. Also, the concentrations of all seven SCFAs were consistently reduced in the model group compared with the control group (*p* < 0.05, *p* < 0.01), and the addition of GRP resulted in a marked enhancement in the concentrations of these SCFAs (Figure 6B–H, *p* < 0.05). Nevertheless, acetic acid, propionic acid, and butyric acid are the principal constituents of SCFAs, and the contents of three SCFAs were significantly lower in the excreta of alcohol-treated mice compared with the normal mice. Studies have shown that propionic acid and butyric acid influence the conversion of fatty acids in the liver. The GRP intervention resulted in an elevation in the butyric acid content. Butyric acid, a fermentation product of intestinal flora, provides energy to the organism and regulates energy and lipid metabolism in the body as a signaling molecule. Furthermore, several studies have demonstrated the role of butyric acid as a substrate for fat synthesis and as a signaling molecule. It regulates fat metabolism by binding to G-protein-coupled receptors or inhibiting the activity of histone deacetylases [65,66]. Excessive ethanol intake can impede the tricarboxylic acid cycle and fatty acid oxidation, affecting lipid metabolism and leading to TG accumulation in the liver and increased blood TG levels [67]. GRP significantly reduced the levels of TC and TG in mice administered alcohol, suggesting a role of GRP in regulating lipid metabolism disorders. The GRP intervention increased propionic acid levels. Further, propionic acid reduced fatty acid levels in the liver [68] and plasma and ameliorated mitochondrial damage in hepatocytes, thus mitigating cellular oxidative stress and apoptosis [69].

Chun et al. [70] and Koh et al. [71] found that SCFAs, including acetic acid, propionic acid, and butyric acid, were significant metabolites providing nutrients to epithelial cells, regulating intestinal immune homeostasis, and modulating the sugar and lipid metabolisms. Propionic acid inhibits hepatic gluconeogenesis [72], whereas both acetic acid and butyric acid reduce lipogenesis [73,74,75,76]. In addition, the administration of SCFAs reduced hepatic steatosis in animal models [77,78]. Acetate is produced by various bacteria such as *Lactobacillus* during fermentation [79]. Butyric acid is the major metabolite produced during *Firmicutes* fermentation [79,80]. Propionic acid is the main metabolite produced during *Bacteroidetes* fermentation [61]. Lower levels of butyrate have been reported in the feces of heavy drinkers and in ethanol-fed mice [81].

Butyrate reduces the NF-κB signaling pathway and increases the upregulation of anti-inflammatory cytokines such as IL-6 by protecting the intestinal barrier [82], thus ameliorating alcohol-induced liver damage. Xu et al. [83] found that propionic acid supplementation protected against ethanol-induced loss of liver performance and liver steatosis in mice. At the same time, treatment with propionic acid alleviated dysfunction of the intestinal epithelial barrier, restored the expression of intestinal mucosal components, inhibited intestinal inflammation, corrected intestinal flora imbalance, and reduced intestinal hyperpermeability in mice with ALD, thereby reducing lipopolysaccharide leakage. In monocytes, butyrate and propionate inhibited the production of LPS, increased the levels of TNF-α and nitric oxide [84], and reduced the level of NF-κB [85]. Furthermore, SCFAs, specifically propionic acid and butyric acid, impacted fatty acid metabolism in the liver [86]. In summary, GRP reduced alcohol-induced gut inflammation. Thus, the gut microbiota altered the production of SCFAs in the liver. GRP promoted the metabolism of SCFAs through the enterohepatic axis, thus protecting against alcohol liver damage.

### 3.8. Prediction of Targeted Metabolic Pathways in ALD

**Figure 7:**
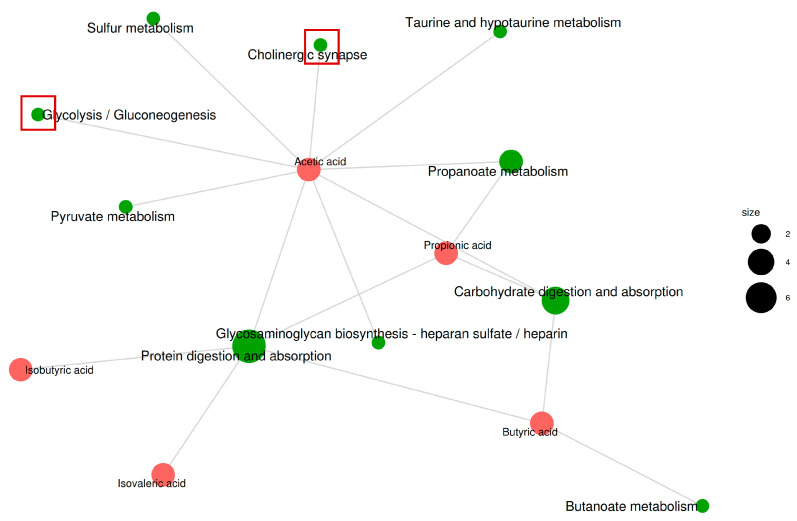
GRP improves the predictive pathways associated with ALD’s effect. Green dots represent metabolic pathways, and other dots represent metabolite molecules. The size of the metabolic pathway point indicates the number of metabolite molecules connected to it, where the higher the number, the larger the point, and the size of the metabolite molecular point indicates the log_2_(FC) value through the gradient change. The colors in the figure represent correlations, with green representing negative correlations and red representing positive correlations. The red boxes are the two main metabolic pathways described below.

We performed a pathway enrichment analysis using the Kyoto Encyclopedia of Genes and Genomes ID of the metabolites to better understand the metabolic changes induced by GRP intervention in alcohol-gavaged mice. The results show that the different metabolite pathways included protein digestion and absorption, carbohydrate digestion and absorption, propanoate metabolism, glycosaminoglycan biosynthesis—heparan sulfate/heparin, cholinergic synapse, taurine and hypotaurine metabolism, pyruvic acid metabolism, sulfur metabolism, butanoic acid metabolism, nicotinic acid and nicotinamide metabolism, phosphonic acid and phosphate metabolism, and glyoxylate and dicarboxylate metabolism (Figure 7). However, propanoate metabolism was the most significant pathway (*p* < 0.01). This was followed by glycosaminoglycan biosynthesis—heparan sulfate/heparin, cholinergic synapse, taurine, and hypotaurine metabolism (*p* < 0.05; Table S1).

Tuma et al. [87] found an association between choline and ethanol metabolism in the liver. The study showed that choline was oxidatively metabolized when stimulated by ethanol. Choline is an essential nutrient, and a decrease in blood choline levels is usually associated with liver steatosis and liver dysfunction [88]. More than 98% of blood and tissue choline is part of the phosphatidylcholine molecule [89]. Phosphatidylcholine is an essential part of animal cell membranes and possesses various desirable physiological activities, including lowering blood fat content, benefiting the liver, and inhibiting atherosclerosis. Liver cell damage caused by alcohol-induced oxidative stress manifests as increased choline levels in the liver tissue of mice with ALD. Oxidative stress can cause lipid peroxidation of the liver cell membrane. Higher levels of choline can damage the cell membrane structure [87,89], resulting in ALD. In this study, it was predicted that the contents of phosphatidylcholine and CDP-choline in mice administered alcohol significantly increased, which was consistent with previous findings [90,91]. The aforementioned situation was reversed by adding GRP. In addition, the expression of more genes in the choline metabolism pathway was upregulated or downregulated by GRP pretreatment. Therefore, it was hypothesized that GRP might regulate choline metabolism, thus affecting ALD.

Glycolysis and the tricarboxylic acid cycle are the principal sources of energy for bodily functions [92]. Increased levels of fumaric acid, which is an intermediate in the tricarboxylic acid cycle, indicate the suppression of glycolysis and ATP metabolism and the enhancement of aerobic metabolism. Pantothenic acid can reduce fatty degeneration, cytoplasmic vacuolization, and necrosis in mice with ALD [93]. Yu et al. [93] found that Coprinus comatus polysaccharide protected against ALD in mice. They also examined metabolomics and gut flora, revealing that ALD was related to glycolysis. *C. comatus* polysaccharide effectively reduced the level of fumaric acid in the ALD group to achieve hepatoprotective effects. The levels of pantothenic acid in the liver tissue abnormally increased. However, the level of pantothenic acid in the *C. comatus* polysaccharide group was reduced, confirming the protective impact of *C. comatus* polysaccharide on ALD in mice. Xing et al. [94] found that excessive alcohol consumption caused liver injury in mice and increased the levels of fumaric acid and pantothenic acid in liver tissue. Based on these findings, we predicted that the levels of fumaric acid and pantothenic acid in the liver tissue of mice fed alcohol increased and caused liver damage. Further, GRP reduced the levels of fumaric acid and pantothenic acid in the liver tissue of mice administered alcohol, resulting in liver protection.

## 4. Conclusions

The findings of this study indicate that GRP could mitigate liver injury in alcohol-administered mice. The addition of GRP reduced liver indices and increased certain serum and liver biochemical indices related to lipid metabolism in these mice. The beneficial effects of GRP might be attributed to its ability to enhance lipid metabolism, suppress inflammatory responses, and alter the structure and abundance of gut microbiota and metabolites in alcohol-fed mice. Also, it ameliorated alcohol-induced intestinal dysbiosis by increasing the abundance of beneficial bacteria and decreasing the abundance of pro-inflammatory bacteria. The results indicate that GRP supplementation increased the contents of SCFAs while modulating the metabolic levels of cholinergic synapses, glycolysis, and other metabolites. These findings provide the foundation for further research into the potential of GRP in improving ALD. GRP has been shown to attenuate alcohol-induced oxidative damage in the liver by modulating the gut microbiota and hepatic metabolic pathways. Consequently, it may serve as a functional component for preventing ALD. Given the limitations of the present study, future studies should assess the effects of different doses of GRP to elucidate other metabolic responses along the gut–hepatic axis and related pathways. Notably, this study emphasizes the significance of examining these results from the perspective of the enterohepatic axis.

## Data Availability

The original contributions presented in the study are included in the article/Supplementary Materials, further inquiries can be directed to the corresponding author.

## Supplementary Materials

The following supporting information can be downloaded at: https://www.mdpi.com/article/10.3390/foods13233733/s1, Figure S1: GRP monosaccharide composition; Figure S2: GRP IR spectrum; Figure S3: UV absorption spectrum of GRP; Figure S4: GPC/SEC analysis of GRP; Table S1: Results of the pathway analysis.
