# Supplementary material for: Green Radish Polysaccharide Prevents Alcoholic Liver Injury by Interfering with Intestinal Bacteria and Short-Chain Fatty Acids in Mice
Source: Foods. 2024 Nov 22;13(23):3733. doi: 10.3390/foods13233733 (PMC11639836; doi:10.3390/foods13233733)
Supplement: Supplementary file 1 [file foods-13-03733-s001.zip › foods-3250393-supplementary.pdf]

Figure. S1 GRP monosaccharide composition.

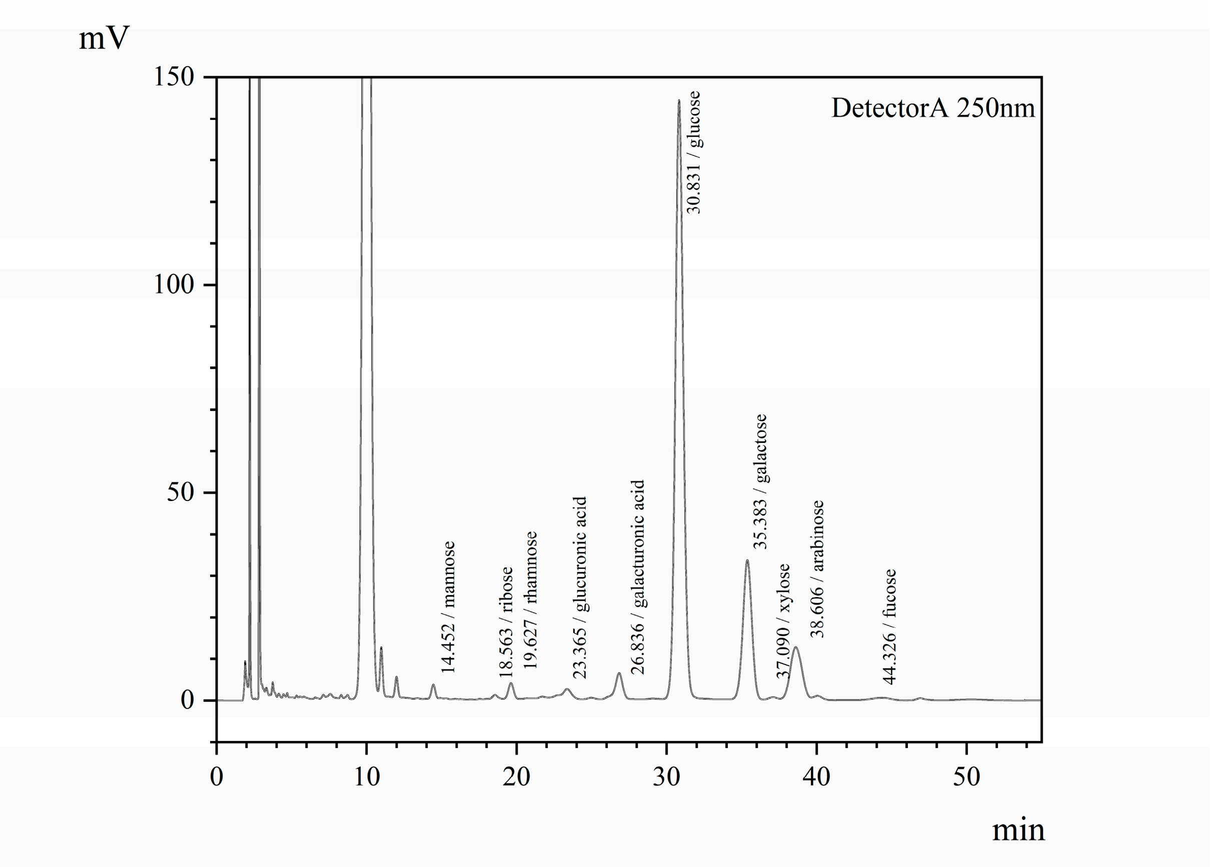

Figure. S2 GRP IR spectrum

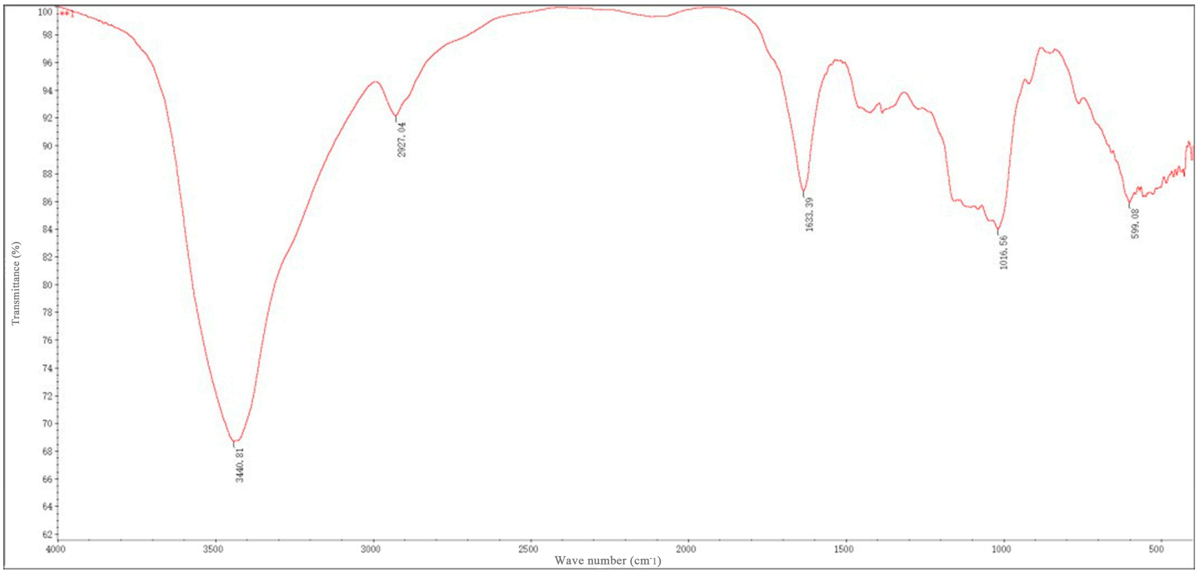

Figure. S3 UV absorption spectrum of GRP

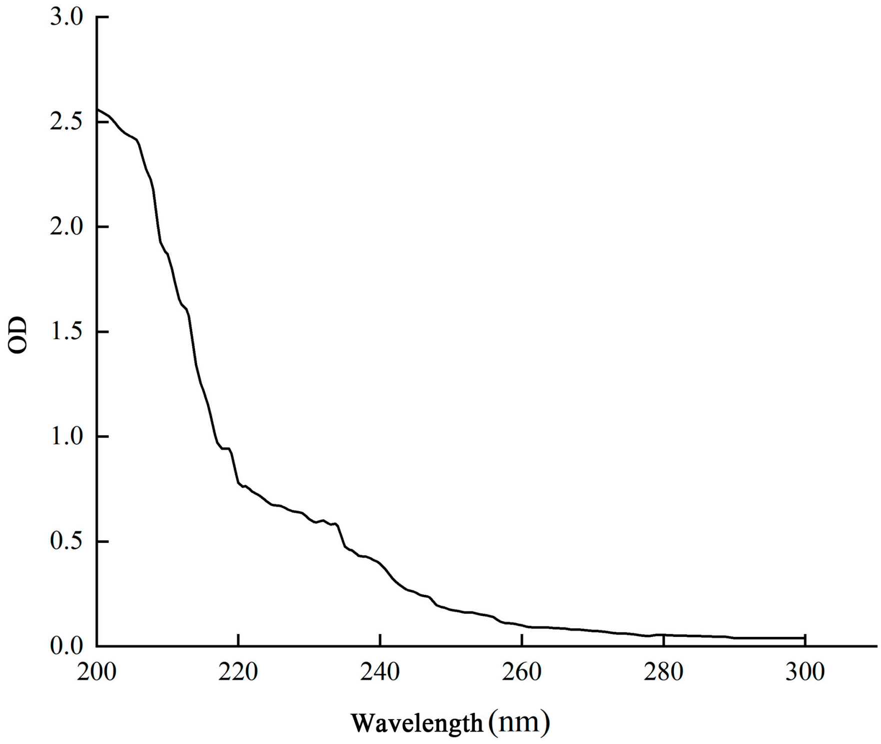

Figure. S4 GPC/SEC analysis of GRP

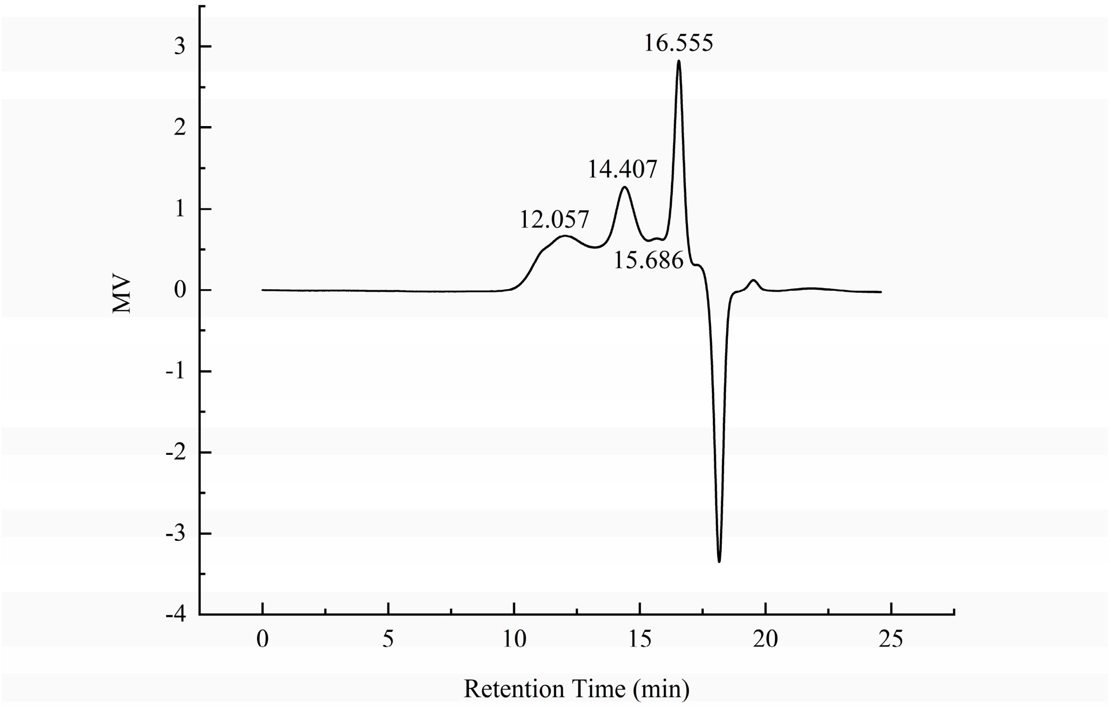

Table. S1 Results of the pathway analysis

pathway_name	Total	Hits	P.value	-Log10(P.value)	FDR	Impact
Protein digestion and absorption	47	5	9.48131E-09	18.47394302	2.57892E-06	0.1064
Carbohydrate digestion and absorption	27	3	1.65129E-05	11.01137058	0.00224575	0.1034
Propanoate metabolism	48	2	0.004116868	5.492662545	0.373262719	0.0385
Glycosaminoglycan biosynthesis - heparan sulfate / heparin	7	1	0.014701214	4.219825191	0.999682564	0.037
Cholinergic synapse	12	1	0.025088309	3.685353309	1	0.1
Taurine and hypotaurine metabolism	22	1	0.045581209	3.088259736	1	0.0294
Glycolysis / Gluconeogenesis	31	1	0.063708016	2.75344489	1	0.0539
Pyruvate metabolism	31	1	0.063708016	2.75344489	1	0.0952
Sulfur metabolism	33	1	0.067695869	2.692730124	1	0.0182
Butanoate metabolism	42	1	0.085461575	2.459688422	1	0.0147
Nicotinate and nicotinamide metabolism	55	1	0.110610507	2.20174019	1	0.0047
Phosphonate and phosphinate metabolism	56	1	0.112520239	2.184622167	1	0.0147
Glyoxylate and dicarboxylate metabolism	62	1	0.123904986	2.088240249	1	0.0103
